# Inactivation of murine norovirus by chemical biocides on stainless steel

**DOI:** 10.1186/1471-2334-9-107

**Published:** 2009-07-07

**Authors:** Thomas Magulski, Dajana Paulmann, Birte Bischoff, Britta Becker, Eike Steinmann, Jörg Steinmann, Peter Goroncy-Bermes, Jochen Steinmann

**Affiliations:** 1 MikroLab GmbH, Norderoog 2, D-28259 Bremen, Germany; 2 TWINCORE – Centre for Experimental and Clinical Infection Research, Department of Experimental Virology, Feodor-Lynen-Strasse 7, D-30625 Hannover, Germany; 3 Institut für Medizinische Mikrobiologie, Universitätsklinikum Essen, Virchowstrasse 179, D-45147 Essen, Germany; 4 Schülke & Mayr GmbH, Robert-Koch Str. 2, D-22851 Norderstedt, Germany

## Abstract

**Background:**

Human norovirus (NoV) causes more than 80% of nonbacterial gastroenteritis in Europe and the United States. NoV transmission via contaminated surfaces may be significant for the spread of viruses. Therefore, measures for prevention and control, such as surface disinfection, are necessary to interrupt the dissemination of human NoV. Murine norovirus (MNV) as a surrogate for human NoV was used to study the efficacy of active ingredients of chemical disinfectants for virus inactivation on inanimate surfaces.

**Methods:**

The inactivating properties of different chemical biocides were tested in a quantitative carrier test with stainless steel discs without mechanical action. Vacuum-dried MNV was exposed to different concentrations of alcohols, peracetic acid (PAA) or glutaraldehyde (GDA) for 5 minutes exposure time. Detection of residual virus was determined by endpoint-titration on RAW 264.7 cells.

**Results:**

PAA [1000 ppm], GDA [2500 ppm], ethanol [50% (v/v)] and 1-propanol [30% (v/v)] were able to inactivate MNV under clean conditions (0.03% BSA) on the carriers by ≥ 4 log_10 _within 5 minutes exposure time, whereas 2-propanol showed a reduced effectiveness even at 60% (v/v). Furthermore, there were no significant differences in virus reduction whatever interfering substances were used. When testing with ethanol, 1- and 2-propanol, results under clean conditions were nearly the same as in the presence of dirty conditions (0.3% BSA plus 0.3% erythrocytes).

**Conclusion:**

Products based upon PAA, GDA, ethanol and 1-propanol should be used for NoV inactivation on inanimate surfaces. Our data provide valuable information for the development of strategies to control NoV transmission via surfaces.

## Background

Human noroviruses (NoVs) are members of the genus *Norovirus *and belong to the family *Caliciviridae *possessing a single-stranded genome without an envelope. NoV is responsible for more than 80% of nonbacterial gastroenteritis in Europe and the United States [1,2]. The most common routes of norovirus transmission are ingestion of contaminated food or water, direct and indirect person-to-person contact and aerosolization of viral particles.

In young children, a long-term shedding for more than one month was observed in 22.6% of those with adequate follow-up [3]. This study in a children's hospital also revealed that 59% of all NoV infections were hospital-acquired. A recent publication confirmed the extensive contamination of environmental surfaces resulting in a prolonged norovirus outbreak [4]. These findings highlight the role of widespread environmental contamination in hospitals and other medical settings. Effective disinfection of surfaces and healthcare equipment is crucial for the prevention of virus transmission. Consequently, inactivation studies simulating practical conditions with different chemical biocides are important to develop surface disinfectants effective against human NoVs.

Due to the lack of a cell culture system for important nosocomial pathogens such as human NoVs, it is necessary to use a surrogate virus which can easily be cultivated in cell culture. In the past, the most suitable surrogate for human NoVs when studying the virucidal activity of chemical disinfectants in suspension and on carriers was the feline calicivirus (FCV) [5-7]. Within the family Caliciviridae, FCV belongs to the genus *Vesivirus *and it is known to be a respiratory pathogen. Therefore, survival and inactivation characteristics may differ from those of human NoVs, which are transmitted by the fecal-oral route.

Recently, propagation of the murine norovirus (MNV) in dendritic cells and macrophages was achieved [8]. Like human NoV, MNV is a member of the genus *Norovirus *and passes through the gastrointestinal tract. Because of this, MNV seems to be a more suitable surrogate for human NoV than FCV [9,10].

This study presents the results of activities of different biocides against the MNV dried on stainless steel to simulate practical conditions. These data should facilitate our understanding of inactivation of human NoV by surface disinfectants.

## Methods

### Preparation of the test virus suspension

Test virus suspension was prepared by infecting RAW 264.7 cells (ATCC TIB-71) with MNV (Berlin 06/06/DE isolate S99, kindly provided by the Robert Koch-Institute in Berlin) by using a multiplicity of infection of 1. Following 2 hours of adsorption at 37°C, the inoculum was replaced by Dulbecco's modified Eagle's medium (DMEM, 4.5 g/l glucose, Lonza Group Ltd., Verviers, Belgium) with 3% fetal calf serum (FCS, low endotoxin, HyClone, PerbioScience, Bonn, Germany). The tissue culture flask was incubated at 37°C and 5% CO_2 _until 70–95% of the cells showed a cytopathic effect (after 1–2 days). The cells were frozen and thawed twice, followed by centrifugation at 1600 g for 10 minutes. The supernatant was aliquoted as test virus suspension and stored at -80°C.

### Chemical biocides

Dilutions of peracetic acid (PAA), glutaraldehyde (GDA) and the different kinds of alcohol (Sigma-Aldrich, Seelze, Germany) were prepared immediately before the inactivation experiments with hard water (300 ppm CaCO_3_, pH 7.0) prepared according to EN 14476:2007-02 and used within 2 hours [11].

### Preparation of virus inoculum

The virus inoculum was prepared by mixing the test virus suspension with the interfering substances according to EN 14476:2007-02 [11]:

a) Nine volumes of test virus suspension were mixed with one volume of 0.3% w/v of bovine serum albumin (clean conditions)

b) Nine volumes of test virus suspension were mixed with one volume of 3% w/v of bovine serum albumin plus 3% v/v washed sheep erythrocytes (dirty conditions).

### Preparation of the carrier

For cleaning, stainless steel discs (20 mm diameter, GK Formblech GmbH, Berlin, Germany) were incubated in a 5% (v/v) Decon^®^90-solution (Decon Laboratories Ltd., Hove, England) for 1 h. Afterwards the discs were rinsed off twice with freshly destilled water for 10 sec, ensuring that the carriers did not dry to any extent, and were then placed in 70% ethanol (v/v) for 15 min. Finally, the carriers were dried by evaporation in sterile petri dishes in a biological safety cabinet.

### Experimental procedure

50 μl of the virus inoculum were pipetted in the middle of each pre-treated carrier and dried in a desiccator with a vacuum of 700–800 mbar for about 30 minutes to reach a constant humidity in all experiments. After drying, the virus-contaminated discs were transferred into 25 ml plastic vial holders (Sarstedt AG & Co. KG, Nümbrecht, Germany), which were previously filled with 0.5 g of glass beads (0.25–0.50 mm diameter, Carl Roth GmbH, Karlsruhe, Germany) to increase virus recovery by mechanical abrasion. 100 μl of the test biocides were then pipetted on the dried virus inoculum and incubated for 5 minutes. Control carriers received 100 μl of hard water instead of the chemical substance. In order to stop the activity of the test substance, 900 μl of culture medium were added immediately at the end of the exposure time. The vials were vortexed directly for 1 min to recover the residual virus before the eluate was diluted to determine viral infectivity. Each test was carried out with 3 replicates (carriers) per concentration of the substance and virus control respectively, with a minimum of two independent experiments.

### Determination of infectivity

Infectivity was determined by endpoint dilution titration onto RAW 264.7 cells. At the end of the exposure time, aliquots of the mixture were taken and diluted immediately. 100 μl of each dilution were placed in eight wells of a sterile 96-well microtiter plate with the permissive RAW 264.7 cells. Plates were incubated for 4 to 5 days and infectivity was analysed by virus-induced cytopathic effect. Virus titres were determined by the method of Spearman and Kärber. Titre reduction was calculated as the difference between the virus titre of the water control (5 min) and the titre of the biocides after 5 min of exposure times and is presented as log_10 _reduction.

The criterion used for virucidal activity of the biocide was a four log_10 _reduction in this study (inactivation of 99.99%).

## Results

### Inactivation of MNV by peracetic acid and glutaraldehyde

**Figure 1:**
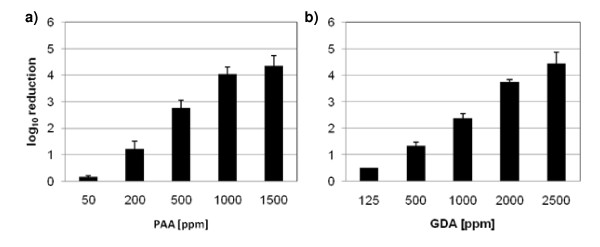
**Effectiveness of peracetic acid and glutaraldehyde against murine norovirus**. **a) **100 μl of peracetic acid [50, 200, 500, 1000, 1500 ppm] or **b) **100 μl of glutaraldehyde [125, 500, 1000, 2000, 2500 ppm] were pipetted on the dried virus inoculums (clean conditions) and incubated for 5 minutes. Results represent the mean log_10 _reduction with standard deviation of three replicates per concentration of the substance of two independent experiments.

Different concentrations of PAA and GDA were evaluated in the carrier test against MNV under clean conditions with a contact time of 5 minutes. Concentrations of 50, 200 and 500 ppm were not able to reduce virus titres sufficiently, whereas PAA in a concentration of 1000 ppm reduced MNV titre by 4.05 log_10_. Testing GDA the concentration had to be increased up to 2500 ppm to achieve a 4 log_10 _reduction in virus titre (Figure 1a and 1b). Lower concentrations were not effective against the test virus on the carriers.

### Inactivation of MNV by different alcohols

**Figure 2:**
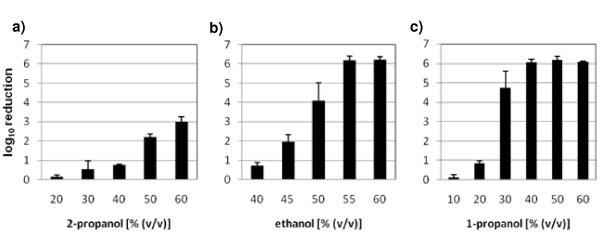
**Effectiveness of different concentrations of 2-propanol, ethanol and 1-propanol against murine norovirus**. **a) **100 μl of 2-propanol [20, 30, 40, 50, 60% (v/v)], **b) **100 μl of ethanol [40, 45, 50, 55, 60% (v/v)] or **c) **100 μl of 1-propanol [10, 20, 30, 40, 50, 60% (v/v)] were pipetted on the dried virus inoculums (clean conditions) and incubated for 5 minutes. Results represent the mean log_10 _reduction with standard deviation of three replicates per concentration of the substance of two independent experiments.

Ethanol, 1-propanol and 2-propanol were tested for their ability to inactivate MNV on stainless steel discs. These different alcohols varied in their capability to inactivate MNV under clean conditions. Whereas the highest used concentration of 60% (v/v) 2-propanol only reduced the viral titre by 3.02 log_10 _within 5 minutes of exposure time (Figure 2a), 50–55% (v/v) ethanol was able to reduce the infectivity of MNV by 4.09 to 6.18 log_10 _(Figure 2b). The most effective alcohol in the carrier test was 1-propanol. 30% (v/v) 1-propanol was sufficient to inactivate 99.99% of MNV after a contact time of 5 minutes. A concentration of 40% (v/v) 1-propanol decreased virus titre on the carrier by an average of 6.04 log_10 _(Figure 2c).

### Influence of soil load on MNV inactivation by different alcohols

**Figure 3:**
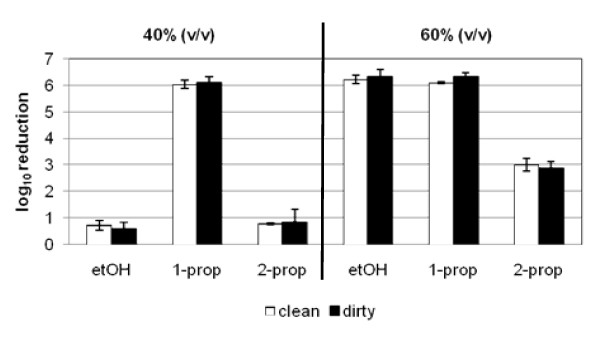
**Comparison of the inactivation properties of different alcohols under clean and dirty conditions**. 100 μl of ethanol [40, 60% (v/v)], 1-propanol [40, 60% (v/v)] or 2-propanol [40, 60% (v/v)] were pipetted on the dried virus inoculums and incubated for 5 minutes. Results represent the mean log_10 _reduction with standard deviation of two replicates per concentration of the substance of two independent experiments.

To compare the influence of soil load, we analyzed the three alcohols for their inactivation properties against MNV under clean and dirty conditions. Reduction factors achieved with the corresponding alcohols in concentrations of 40% (v/v) and 60% (v/v) under clean conditions were comparable to those measured under dirty conditions (Figure 3).

## Discussion

Norovirus infections spread rapidly particularly in senior residences, hospitals, hotels, schools and even cruise ships. Apparently, contaminated surfaces may play an important role in the spread of human NoV [12-15]. In a rehabilitation centre, the protracted course of norovirus-induced gastroenteritis was also connected to an environmental contamination [16]. Therefore, measures for the prevention and control of NoV transmission should include surface disinfection with products highly effective against this viral pathogen.

Many surface cleaners in the household are based on 0.5% hypochlorite. It was shown by reverse transcriptase polymerase chain reaction (RT-PCR) for human NoVs that the cleaning of fecally contaminated surfaces with a cloth soaked in detergents or treatment with 0.5% hypochlorite alone failed to eliminate human NoVs. The virus could still be detected on up to 28% of the surfaces by RT-PCR. Only wiping the surface with a cloth soaked in detergents followed by applying a combined hypochlorite/detergent treatment was successful to achieve a sufficient efficacy against human NoVs [17].

Since there is only limited knowledge about human NoV inactivation on surfaces, it was the aim of this study to evaluate the virucidal properties of some often-used chemical ingredients of surface disinfectants in a standardized test procedure. The importance of carrier-based methods to test efficacy of disinfectants against vertebrate viruses has been stressed [18].

Meanwhile, carrier test methods including a ring trial launched under the initiative of the Organisation for Economic Cooperation and Development have been published [19-21]. In our study the protocol used followed the draft of the European Committee for Standardization TC 216 with 0.03% BSA and 0.3% BSA plus 0.3% sheep erythrocytes as interfering substances without mechanical action [22].

The absence of a cell culture assay for human NoV makes it necessary to introduce a reliable surrogate virus when studying the environmental persistence and efficacy of chemical disinfectants. Because FCV can be propagated in cell culture, it has been extensively studied in inactivation studies in the past [5,23]. In contrast to FCV, MNV is transmitted by the fecal-oral route, thus making this virus a promising surrogate for human NoVs. Consequently, stability and inactivation by alcohol-based hand rubs and commercial products used in veterinary medicine for surface disinfection have been successfully tested with MNV in suspension and carrier tests [24-26].

In this study, MNV showed a remarkable stability while drying. The difference in the virus titre before and after drying on the carrier did not exceed 0.35 log_10 _(data not shown in table). In contrast, the poliovirus titre decreased by about 3 log_10 _during the drying process [27].

In general, non-enveloped viruses can persist for approximately up to two months on inanimate surfaces [28]. For example, reovirus was able to survive on polyvinyl chloride carriers for a period of 30 days dried in organic matrix [29].

MNV can persist in various environmental conditions. After 40 days at -20°C and 4°C only a < 2 log_10 _reduction was observed [30]. This study also revealed that MNV survived better in a stool suspension than on the surface of gauze or diaper material.

Recently, the stability of MNV mixed with artificial feces on stainless steel coupons was studied [25]. In this study, only a minimal loss of infectivity was measured at pH 2, whereas FCV was rather unstable at pH values lower than 3.

In this study we show that PAA and GDA were able to reduce the titre of MNV under clean conditions about more than 4 log_10 _within 5 minutes of exposure time. When testing PAA a concentration of 0.1% (1000 ppm) and when testing GDA a concentration of 0.25% (2500 ppm) was needed to inactivate 99.99% of the viruses. These results were confirmed by the ability of two commercial products based upon GDA and PAA to inactivate MNV [26].

In tests with other viruses simulating practical conditions, 2.0% GDA was able to inactivate HIV, rotavirus and HAV on carriers within one minute [31-33]. Additionally, reovirus embedded in an artificial test soil was inactivated by GDA within one minute, whereas a concentration of 0.1% took 12 minutes to inactivate this virus completely [29].

Generally, besides PAA and GDA, chlorine-based products are often recommended to inactivate non-enveloped viruses on contaminated inanimate surfaces. Testing nonporous and porous surfaces, 20 to 200 ppm of hypochlorous acid solution reduced the titres of human NoV and MNV by 3 log_10 _within a contact time of 10 minutes [34].

In further experiments we analysed the virucidal properties of ethanol, 1-propanol and 2-propanol against MNV in the quantitative carrier test under clean conditions with concentrations from 10 to 60% (v/v). Since results from the suspension test with the MNV where ethanol in a concentration of 60% (v/v) to 80% (v/v) was more effective than 1-propanol followed by 2-propanol (unpublished), we expected ethanol to be the most effective virucidal agent, followed by 1- and 2-propanol. Surprisingly, 1-propanol was the most effective alcohol to inactivate MNV on the carrier under clean conditions. Interestingly, a concentration of only 30% (v/v) 1-propanol was sufficient to inactivate 99.99% of the virus. In comparison, the two other alcohols were completely ineffective at this concentration. Ethanol achieved a similar effectiveness only at 50–55% (v/v), while 2-propanol at a concentration of 60% (v/v) did not reach a 4 log_10 _reduction. These results are in contrast to data with FCV. Here, 2-propanol inactivating 99% within one minute at 40 to 60% was found to be more effective than ethanol (6).

Approaching practical conditions, the soil load was increased from 0.03% BSA (clean conditions) to 0.3% BSA plus 0.3% sheep erythrocytes (dirty conditions). Previous results of suspension tests or carrier assays had shown that a higher organic load resulted in a decreased effectiveness of the biocide [35-37]. Interestingly, our results could not confirm these findings. There was no difference in log_10 _reduction detectable between clean and dirty conditions. All three alcohols tested showed similar reduction factors at 40% (v/v) and 60% (v/v) with 0.03% BSA as well as with 0.3% BSA plus 0.3% erythrocytes. At the tested concentration of 40% (v/v) 1-propanol was highly active against MNV after an exposure time of 5 minutes by reaching a reduction of 6 log_10_, whereas ethanol and 2-propanol could not inactivate the virus sufficiently. The effectiveness of ethanol and 2-propanol could be enhanced by increasing the concentration of alcohol. At an alcohol concentration of 60% (v/v) ethanol reached an efficacy comparable to 1-propanol, whereas 2-propanol was not capable of reducing the virus titre by more than 3 log_10 _at the same concentration.

In summary, our data indicate that 1-propanol was the most effective alcohol to inactivate MNV on stainless steel discs within 5 minutes of contact time. Independent of the amount of soil load, even a concentration of 30% (v/v) 1-propanol had the capability to decrease the virus titre by ≥ 4 log_10 _within 5 minutes.

In further studies, it would be interesting to compare the inactivating properties of these biocides against MNV on other carriers often used in hospitals and other medical settings.

## Conclusion

To interrupt the transmission of human NoV it is necessary to use highly effective surface disinfectants tested under practical conditions. Our results show that MNV as a surrogate for human NoV is inactivated under clean conditions by PAA and GDA as well as by the two alcohols ethanol and 1-propanol on stainless steel discs after 5 minutes of exposure time, whereas 2-propanol only achieved insufficient effectiveness. The effective concentrations of the chemical biocides examined provide an informative basis when evaluating these substances as possible active ingredients of surface disinfectants with short contact times.

## Competing interests

Dr. P. Goroncy-Bermes is employed by Schülke & Mayr, Norderstedt, Germany

## Authors' contributions

All authors contributed to the conception and analysis of data. TM carried out the experiments. All authors are involved in drafting the manuscript. All authors have read and approved the final version of the manuscript.

## Pre-publication history

The pre-publication history for this paper can be accessed here:

http://www.biomedcentral.com/1471-2334/9/107/prepub
